# Manganese-enhanced MRI of the myocardium

**DOI:** 10.1136/heartjnl-2019-315227

**Published:** 2019-07-23

**Authors:** Nick B Spath, Gerard Thompson, Andrew H Baker, Marc R Dweck, David E Newby, Scott I K Semple

**Affiliations:** 1 BHF/University Centre for Cardiovascular Sciences, University of Edinburgh, Edinburgh, UK; 2 Edinburgh Heart Centre, Royal Infirmary of Edinburgh, Edinburgh, UK; 3 Edinburgh Imaging, University of Edinburgh, Edinburgh, UK; 4 Centre for Clinical Brain Sciences, University of Edinburgh, Edinburgh, UK

**Keywords:** MEMRI, manganese-enhanced MRI, viability

## Abstract

Gadolinium-based contrast media are widely used in cardiovascular MRI to identify and to highlight the intravascular and extracellular space. After gadolinium, manganese has the second highest paramagnetic moment and was one of the first MRI contrast agents assessed in humans. Over the last 50 years, manganese-enhanced MRI (MEMRI) has emerged as a complementary approach enabling intracellular myocardial contrast imaging that can identify functional myocardium through its ability to act as a calcium analogue. Early progress was limited by its potential to cause myocardial depression. To overcome this problem, two clinical formulations of manganese were developed using either chelation (manganese dipyridoxyl diphosphate) or coadministration with a calcium compound (EVP1001-1, Eagle Vision Pharmaceuticals). Preclinical studies have demonstrated the efficacy of MEMRI in quantifying myocardial infarction and detecting myocardial viability as well as tracking altered contractility and calcium handling in cardiomyopathy. Recent clinical data suggest that MEMRI has exciting potential in the quantification of myocardial viability in ischaemic cardiomyopathy, the early detection of abnormalities in myocardial calcium handling, and ultimately, in the development of novel therapies for myocardial infarction or heart failure by actively quantifying viable myocardium. The stage is now set for wider clinical translational study of this novel and promising non-invasive imaging modality.

## Introduction

Cardiovascular MRI is an essential tool in the diagnosis of a range of cardiovascular diseases. Significant advances in expertise, technical capabilities and accessibility mean cardiac MRI is a routine clinical assessment. It is the accepted gold-standard method for quantification of myocardial volumes, mass and function, with an increasing role in valvular heart disease. Contrast imaging with gadolinium delayed-enhancement MRI (DEMRI) allows quantification of myocardial fibrosis, especially for assessment of myocardial viability in ischaemic cardiomyopathy. However, DEMRI only allows extracellular space imaging, describing infarcted or scarred tissue, from which myocardial viability is inferred. By comparison, manganese-enhanced MRI (MEMRI) directly provides intracellular contrast of viable myocardium. Furthermore, long-term safety questions have been raised regarding some gadolinium formulations.^1^ This review explores the potential of novel manganese-based contrast agents in MRI, providing a synopsis of published work since the comprehensive review by Wendland 15 years ago,^2^ and discussing future potential clinical applications of MEMRI of the myocardium.

## Historical perspective: manganese as a contrast agent

Paramagnetic compounds have long been used in MRI to modify relaxivity, providing contrast enhancement for tissue characterisation. Gadolinium-based chelates dominate current clinical use. Their size and physical properties prevent them crossing intact cell membranes and they initially disperse equally throughout extracellular space. Tissues with abnormal extracellular dynamics clear gadolinium more slowly, resulting in reduced T1 in pathological regions.

Manganese was the first element used as an MRI contrast agent in vivo, with significant cardiac, hepatic and renal uptake causing marked shortening of T1 relaxation.^3^ Manganese is an essential trace element required as a cofactor and enzyme activator for bone development, neurological, immunological and endocrine function, and is also key to protecting mitochondrial membranes from oxidative stress through superoxide dismutase.^4^ The impact of manganese-protein binding on proton relaxation was first studied in 1962 and in 1973, Lauterbur used manganese to image differential T1 relaxation using nuclear magnetic resonance.^5^ Manganese has key properties of interest for MRI. First, its paramagnetism reduces T1 relaxation of water, enabling contrast in tissues where it accumulates, providing excellent anatomical delineation.^6^ Second, it is a calcium analogue, crossing myocardial L-type voltage-gated calcium channels and sodium-calcium exchangers.^7^ Third, its natural occurrence in vivo as an essential trace element makes its safety and toxicity profile more attractive than gadolinium which has no known mammalian biological role.^8^

Manganese contrast agents offer potential in numerous MRI platforms having been applied to preclinical neuronal assessment,^6^ lymphocyte and stem cell tracking,^9^ pancreatic beta cell activation^10^ and the evaluation of myocardial viability in ischaemia.^11^ Manganese radiotracers have also been explored in positron emission tomography.^12^ Interest in the field is evidenced by a steady increase in publications over the past 15 years.

## Calcium-dependent myocardial contrast imaging

Unlike gadolinium, manganese provides intracellular myocardial contrast imaging through calcium handling. During myocardial contraction, calcium enters cardiomyocytes predominantly through voltage-gated L-type calcium channels triggering calcium release from the sarcoendoplasmic reticulum (excitation–contraction coupling).^13^ In diastole, calcium is actively transported into sarcoendoplasmic reticulum by calcium ATP (SERCA2a), in addition to passage into the extracellular space via the sodium–calcium exchanger and mitochondrial uptake.^13^ Alterations in calcium handling by protein dysfunction and defective regulatory mechanisms impair the myocyte’s ability to increase and to decrease intracellular calcium concentrations, impacting systole and diastole accordingly.^14^ Its fundamental role in excitation–contraction coupling makes calcium handling central to pathophysiological and adaptive mechanisms of defective contractile function and impaired relaxation in cardiomyopathy. Several studies have demonstrated reduced sarcoendoplasmic reticulum calcium storage and release, with decreased SERCA2a and upregulated sodium–calcium exchanger activity.^15^ Altered expression and activity of voltage-gated L-type calcium channels in heart failure and hypertrophy observed in several studies are incompletely understood,^16^ although highlight the centrality of calcium handling to cardiomyocyte dysfunction.

The major interest in manganese lies in its biological functionality. As a calcium analogue, manganese is taken up avidly by voltage-gated calcium channels into cells with active calcium handling. Correspondingly, its uptake is reduced or absent in stunned or infarcted tissue, respectively.^17^ The resulting reduction of T1 relaxation times in tissue with functioning calcium handling hypothetically enables selective identification of viable cardiomyocytes. While DEMRI with gadolinium demarcates tissues with pathological extracellular space and scar, MEMRI has the ability to label viable myocardium directly, marking non-viability with reduced manganese uptake by reduced or absent T1 shortening.

## Overcoming toxic potential of manganese

Most human exposure to manganese occurs naturally as it is present in nearly all diets, with stable levels maintained by homoeostatic mechanisms almost regardless of intake.^18^ Naturally occurring deficiency is rare, but toxicity from overexposure are recognised, often following environmental or occupational contact.^19^ With toxic exposure, manganese can accumulate in the striatum and globus pallidus, manifesting as headache and emotional lability, with parkinsonian extrapyramidal symptoms and gait-disturbance (collectively termed manganism).^20^ In its early development, cardiovascular toxicity occurred in animal studies with administration of manganese chloride because it competed too strongly with myocardial calcium uptake, causing myocardial depression, hypotension and cardiac arrest.^21^ MEMRI remained underdeveloped for many years due to initial safety concerns and concomitant advances in gadolinium-based imaging techniques.

Substantial subsequent work has sought to overcome potential manganese toxicity. For intracellular myocardial imaging, manganese must be freely circulating for cardiomyocyte uptake. Unlike agents which strongly bind manganese without dissociating, formulations have been developed which negate risks of toxicity while simultaneously maintaining the desired magnetic and kinetic properties for myocardial imaging. To date, two different methods have been employed producing distinct clinical-grade agents, both with excellent safety profiles: (1) chelation, producing lower effective circulating molar dose of manganese, and (2) coadministration with calcium, competing with manganese at the sarcolemmal membrane to reduce cardiotoxicity. The two most clinically relevant formulations are discussed in table 1.

**Table 1:** Status of manganese contrast agents with clinical experience in myocardial imaging

Contrast agent	Proposed clinical dose (μmol/kg)	Clinical trial/licensing status	Commercial availability	Barriers to clinical application	Currently recruiting or yet-to-report cardiac clinical trials
Chelated manganese: MnDPDP	5	Previously licensed in EU Phase III clinical trials complete	Previously available as Teslascan*, withdrawn due to lack of demand	No current clinical production	Manganese-enhanced MRI (MEMRI) of the myocardium (NCT03607669)
Non-chelated manganese:					
MnCl_2_†	5	Not licensed or undergoing clinical trial	Not available	Significant cardiotoxic potential in cardiac patients	None
EVP1001-1	1–10	Phase II clinical trials complete	Not available	Currently undergoing further clinical trial Toxicity profile in cardiac patients not well established	Clinical Trial of MEMRI to assess peri-infarct Injury (NCT02933034) Efficacy of EVP1001-1 (see more) in the Assessment of myocardial viability in patients With cardiovascular disease (NCT01989195)

*Marketing authorisation holder: GE Healthcare AS.

†No current plans for routine clinical use at time of writing due to toxicity profile.

EU, European Union; EVP1001-1, Eagle Vision Pharmaceutical; MnCl_2_, manganese chloride; MnDPDP, manganese dipyridoxyl diphosphate.

### Manganese dipyridoxyl diphosphate

Unlike gadolinium chelates designed not to dissociate, dipyridoxyl diphosphate chelation allows manganese to uncouple and circulate as a protein-bound complex, with the majority of manganese cleared through hepatobiliary excretion (<20% renal excretion).^22^ Following administration, biotransformation of manganese dipyridoxyl diphosphate (MnDPDP) occurs by dephosphorylation and transmetallation with zinc, facilitating MRI-detectable intracellular manganese uptake as demonstrated in vitro where tissue uptake and renal clearance occur rapidly.^23^ These findings have been reinforced in subsequent animal^24^ and human studies.^25^ Administering manganese in this way successfully mitigates toxicity while retaining crucial paramagnetic and biological properties for adequate intracellular MRI contrast. In phases I and II trials, MnDPDP was well tolerated, with minor and transient symptoms of flushing, headache and nausea reported in a minority of recipients with more serious adverse events occurring rarely.^26^ MnDPDP is an established clinical agent with utility in the diagnosis and staging of hepatobiliary neoplasms,^27^ which was superceded by gadolinium-based agents for upper gastrointestinal indications, market authorisation being withdrawn for economic reasons.

### EVP1001-1

Eagle Vision Pharmaceuticals, (Downingtown, USA) has developed patented an alternative method to chelation of countering adverse effects from manganese, reconstituting magnesium gluconate with a source of calcium.^28^ In this form, manganese has a short plasma half-life, rapid myocardial uptake and slow clearance, with little redistribution.^29^ At the time of writing, the precise details of the formulation are not publicly available with clinical trials ongoing. Limited available data from one clinical study (NCT01989195) did not report any adverse side effects, where oral antiemetic was given prior to administration of the agent.

## Preclinical studies with  MEMRI

The myocardial uptake and dynamics of manganese are reviewed in detail elsewhere.^2^ In brief, evaluation of dose–response curves initially suggested a linear relationship between manganese chloride and myocardial signal intensity,^30^ but subsequent studies have shown that the relationship between manganese dose and change in relaxivity is more accurately described by two linear uptake patterns with a plateau phase at very high doses, likely attributable to rate-limiting renal and biliary excretion.^31^ Importantly, the dose–response relationship appears linear up to a dose of approximately 165 μmol/kg, over 30 times the clinical dose proposed for cardiac imaging. While calcium influx is predominantly dependent on voltage-gated calcium channels, efflux from the myocardium appears more related to sodium–calcium exchangers. Thus, the rate of efflux increases exponentially with increasing manganese dose and is reduced by a sodium–calcium exchange inhibitor.^32^ These comprehensive preclinical dose–response studies support the feasibility of quantitative analysis of the effect of manganese in the heart.

### Myocardial infarction and MEMRI

**Figure 1:**
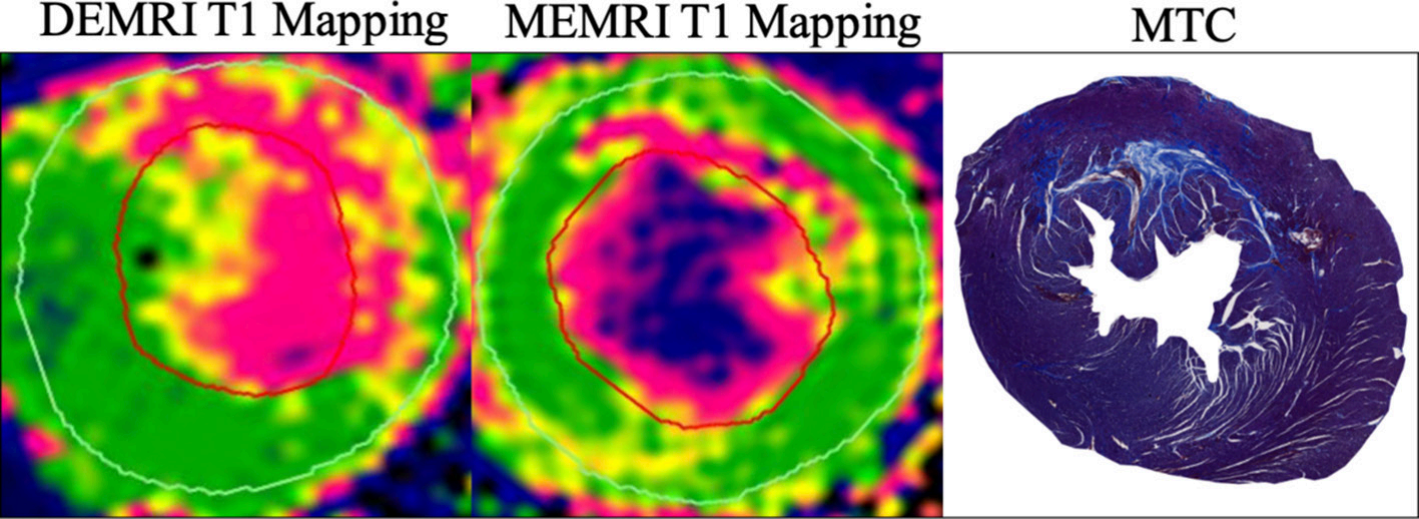
Preclinical manganese-enhanced MRI (MEMRI). MEMRI T1 mapping of acute myocardial infarction in a preclinical model of myocardial infarction in rat, with MnDPDP. Colour maps are configured to show infarct (pink) and remote myocardium (green) with an intermediate peri-infarct zone (yellow). Note the visibly smaller infarct size with MEMRI compared with DEMRI, more in line with histological description of infarct with Masson’s trichrome (MTC). DEMRI, delayed-enhancement MRI; MnDPDP, manganese dipyridoxyl diphosphate.

Various studies have investigated quantification of myocardial infarction with MEMRI. While most use coronary artery ligation or occlusion models, one study demonstrated that MEMRI could differentiate normal from infarcted myocardium in a model of chronic cryoinjury, showing agreement in percentage of left ventricular infarction between MEMRI and DEMRI.^33^ Interestingly, the investigators found no difference in signal intensity in regions of myocardium during infusion of manganese, only observing the difference after termination of the infusion, suggesting that important uptake dynamics follow the initial perfusion phase. In permanent coronary artery occlusion myocardial infarction, the developers of EVP1001-1 reported a marked difference in longitudinal relaxation between infarcted and normal tissue, persisting for at least 1 hour.^34^ The investigators did not quantify infarct size, simply reporting qualitative agreement between extent of infarction between EVP1001-1 and histopathology, inferring clinical potential. Agreement between MEMRI infarct quantification using both MnDPDP and EVP1001-1 has been shown 12 weeks postinfarction by coronary artery ligation, with DEMRI overestimating infarct size acutely perhaps reflecting peri-infarct tissue oedema (figure 1).^35^

### Myocardial viability and MEMRI

Beyond infarct quantification, MEMRI has the potential to assess myocardial viability. Using MnDPDP in acute ischaemia-reperfusion injury by coronary artery ligation, MEMRI assessment of myocardium in vivo correlated with equivalent ex vivo MEMRI images^17^ as well as areas of regional wall motion abnormality.^36^ Interestingly, areas of MEMRI enhancement were greater than histopathological quantification of infarcted myocardium,^17^ suggesting that stunned myocardium also exhibits decreased manganese uptake.

The timing of manganese administration relative to injury has also been assessed. In a coronary artery ligation model of ischaemia reperfusion where MnDPDP was administered acutely with vessel occlusion, reduced MEMRI enhancement correlated with area at risk by methylene blue but interestingly not when administered >6 hours postreperfusion, when reduced MEMRI enhancement correlated with histopathological infarction.^37^ This implies that MEMRI enhances myocardium within injured but not infarcted myocardium and could be an important biomarker of viability. More recent studies have employed this principle using DEMRI and MEMRI together to identify peri-infarct viability, defined as regions which dual-enhance with both gadolinium and manganese.^38^ In the field of myocardial stem cell therapy, this approach has been reported to show successful engraftment in peri-infarct regions following ischaemia reperfusion by endovascular occlusion,^39^ highlighting the potential applications of MEMRI in the development of novel myocardial regenerative therapies.

### Myocardial contractility and MEMRI

Dobutamine is a β_1_-adrenoceptor agonist, increasing myocardial calcium uptake through protein kinase A-mediated pathways and voltage-gated calcium channels by upregulating cyclic AMP activity.^40^ MEMRI can detect pharmacological up-regulation and down-regulation of calcium-channel activity, using dobutamine and diltiazem, respectively,^41^ demonstrating potential to assess the rate of myocardial calcium influx. In addition, dobutamine stress clearly enhances the T1 shortening effect of manganese on myocardium, independent of the rate of administration of contrast agent, suggesting increased manganese influx^42^ with concomitant increase in efflux with dobutamine stress.^32^ Furthermore, improved contrast between normal and ischaemic myocardium with greater reduction in remote myocardial T1 during both dipyridamole and dobutamine stress has been reported with EVP1001-1 in both acute and chronic models of ischaemia.^29^ The authors hypothesise that its rapid uptake kinetics mean EVP1001-1 alone, and not chelated MnDPDP, is suitable for stress imaging, although this has not been formally studied.

### Altered calcium handling and MEMRI

Beyond detection and quantification of myocardial infarction and viability, there is a major clinical interest in whether MEMRI can describe more subtle alterations in calcium handling in various myocardial pathologies. In ischaemic remodelling, one study has looked at changes in T1 of the remote myocardium between early and late post-infarct time points.^35^ The authors observed a weak but significant correlation between impairment of left ventricular ejection fraction and change in T1 over time, suggesting greater manganese uptake in remote remodelling myocardium of more severe ischaemic cardiomyopathy phenotypes. Animal models of non-ischaemic cardiomyopathy are limited but MEMRI in myocardial hypertrophy has been evaluated, with clear reduction in R1 of myocardium following pharmacological induction of hypertrophy compared with normal myocardium, mimicking hypertensive heart disease.^43^ The cellular mechanisms underlying this have yet to be explored in detail, but it points to important potential of MEMRI for non-invasive detection of calcium dysregulation.

## Clinical studies with MEMRI

Despite extensive preclinical data, there are comparatively little clinical cardiac MEMRI data, with most using chelated agents. However, short-term cardiac safety of low-dose intravenous manganese chloride (5 μmol/kg) in 15 healthy volunteers was explored, with T1 shortening properties commensurate with other data, without myocardial depression or toxicity.^44^ Focussing only on short-term cardiac safety in healthy volunteers, further conclusions are limited. At the time of writing, there are no ongoing clinical trials investigating of manganese chloride in humans.

**Figure 2:**
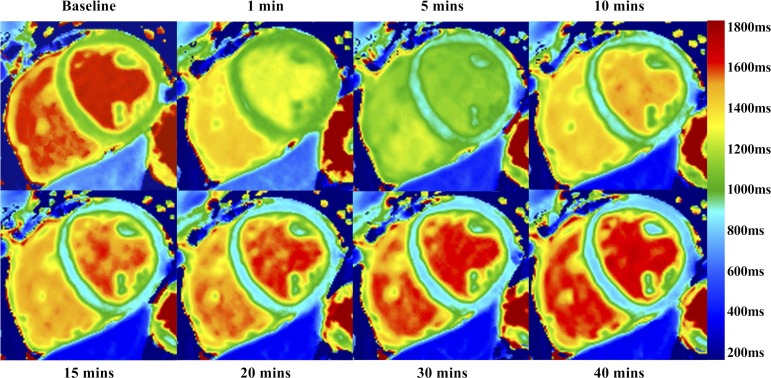
Clinical manganese-enhanced MRI (MEMRI). MEMRI T1 mapping in healthy myocardium with manganese dipyridoxyl diphosphate. Rapid reduction in T1 is seen in the blood pool, followed by rapid normalisation by 40 min. In contrast, the T1 value of remote myocardium shows steady and sustained reduction throughout the imaging time period.

**Figure 3:**
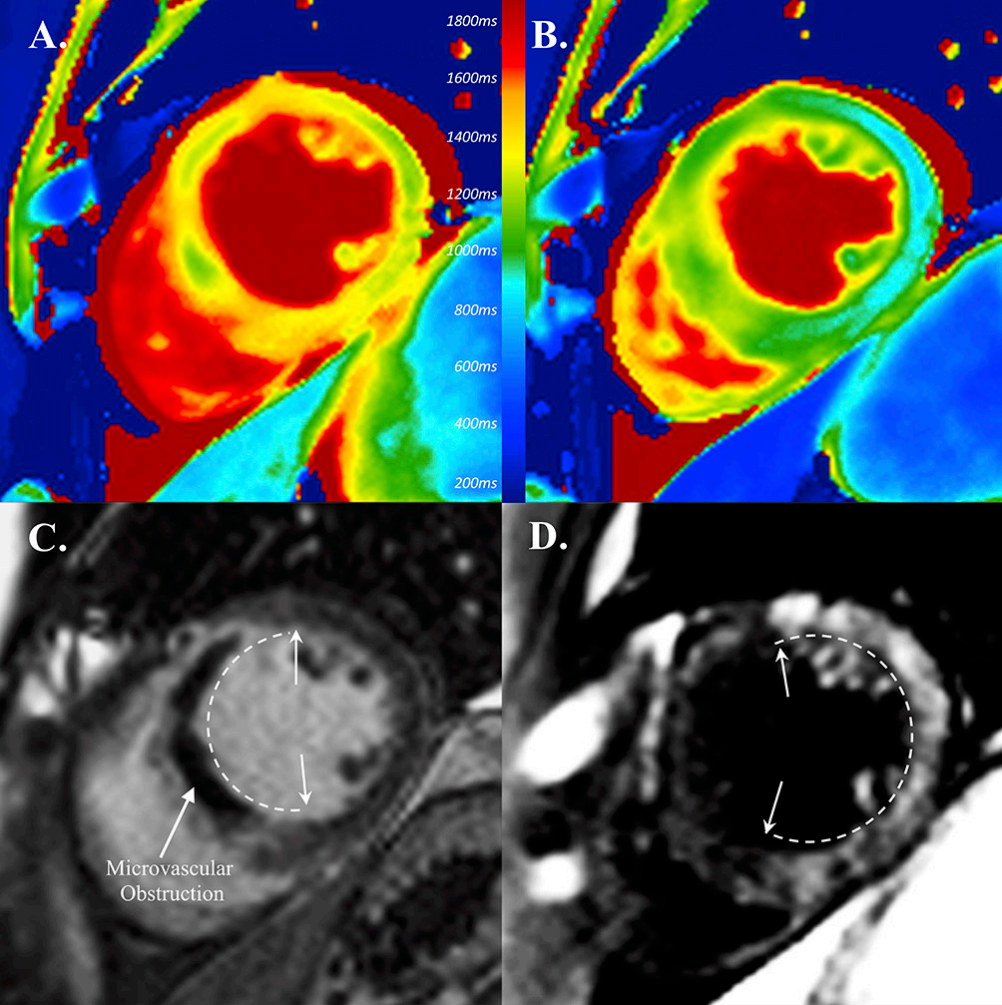
Clinical manganese-enhanced MRI (MEMRI) in a patient with myocardial infarction. Clinical tissue characterisation acutely post-myocardial infarction with native T1 mapping (A), MEMRI T1 mapping (B), gadolinium delayed-enhanced MRI (C) and manganese enhancement (D). Note shortened T1 in remote myocardium on MEMRI compared with native T1 mapping (A, B). Inversion recovery images demonstrate shortening in infarct with gadolinium (C) and in remote myocardium with manganese (D) (extent of enhancement indicated by arrows and dashed lines).

Building on extensive preclinical data, Skjold *et al* have demonstrated the effectiveness of MnDPDP in imaging human myocardium.^45^ They reported consistent reduction in T1 relaxation (34%–46%) in healthy myocardium 30 min after MnDPDP administration, observing only marginal differences in T1 relaxation rate between doses of 5, 10 and 15 μmol/kg. In a subsequent study, the investigators modelled the change in myocardial manganese concentration and unidirectional influx over time with different administration rates.^46^ Only minor differences in myocardial manganese uptake rate were observed between administration over 5 or 30 min, seen predominantly in the early infusion phase with similar overall concentrations in both groups. In patients with myocardial infarction, MEMRI could discriminate between infarcted and remote myocardium, with lower T1 in non-infarcted myocardium.^47^ Beyond acute myocardial infarction, limited work has been done in chronic cardiomyopathy. To date, the data consist of a single conference abstract evaluating MEMRI in a small number of patients with severe chronic cardiomyopathy.^48^ Precontrast T1 quantified by turbo-spin echo was higher than in healthy subjects, but still showed a sharp reduction after MnDPDP administration. Dramatically, lower T1 values were observed in a subset of patients with signal enhancement in dyskinetic regions, suggesting greater calcium-channel activity. These limited speculative findings require corroboration in validated clinical studies. One study is currently recruiting patients with ischaemic and non-ischaemic cardiomyopathy (NCT03607669), projecting a preliminary report later this year (figures 2 and 3, table 1).

In addition to extensive animal studies, EVP1001-1 has been evaluated in phase I and phase II clinical trials. The phase II clinical trial of this agent (NCT00340925) remains unpublished at the time of writing. A follow-up study (NCT01989195) has reported preliminary results in a small cohort of patients (n=6) with previous myocardial infarction which, although yet to be published, suggest minimal variation in heart rate and QRS duration with manganese, as well as significantly different quantification of myocardial infarction for MEMRI compared with DEMRI (18.25±1.7 and 41%±11.4%, respectively). As with MnDPDP, clinical studies of EVP1001-1 have been undertaken to evaluate myocardial ischaemia and viability, yet to report (NCT02933034, NCT01989195, table 1).

## Safety concerns over gadolinium

Gadolinium-based contrast agents have been associated with nephrogenic systemic fibrosis. However, this has largely been eliminated by restricted use of these agents in renal disease, and avoidance of non-ionic linear agents. More recent concerns have arisen around neurological gadolinium deposition. Postmortem analysis has demonstrated neurological accumulation in patients without severe renal disease who had undergone intravenous gadolinium contrast administration, with undetectable levels in control subjects.^1^ This has led to the suspension of some linear gadolinium agents from the European market, with further work needed to clarify the propensity of macrocyclic agents to accumulate in the central nervous system and to establish if this is associated with clinical sequelae. These safety concerns highlight our current dependence on gadolinium and the relative lack of alternatives.

## Future directions

Myocardial imaging with MnDPDP relies on dissociation of manganese, whereby MEMRI is not easily applied to extracellular or intravascular blood pool imaging. However, recent early clinical work shows the potential of a manganese-based contrast medium to provide contrast-enhanced magnetic resonance angiography. Being highly resistant to dissociation, it provides similar angiographic results to gadolinium equivalents with the benefit of avoiding gadolinium administration.^49^

Recent studies have revisited the potential utility of manganese radiotracers.^50^ While these have predominantly focused on applications in neuronal and pancreatic tissues, there is equal scope for application to cardiac imaging. This is especially relevant with the recent advances in hybrid positron emission tomography/MRI techniques, with potential for manganese radiotracers to provide detailed structural and functional myocardial tissue characterisation.

As reviewed here, the ability of MEMRI to detect altered calcium handling is potentially ground breaking in the field. Unlike DEMRI, MEMRI offers exciting potential to diagnose and to quantify myocardial viability post-infarction, rather than infer likelihood of functional recovery based on anatomical scar assessment, offering more accurate prediction of disease progression. Beyond ischaemia, improved understanding of how non-ischaemic cardiomyopathic processes impact cellular calcium handling mechanisms may enable earlier diagnosis, improved prognostication and monitoring as well as guide novel therapeutic strategies.

Since the removal of Teslascan from the drug product list by the Food and Drug Administration (FDA) in 2003 and its withdrawal from the European market in 2012 by the marketing-authorisation holder, no manganese contrast medium has been clinically available. The reasons for this are multifactorial, involving lack of clinical demand due to safety and utility of gadolinium-based agents, as well as lack of large scale clinical translational MEMRI study despite promising clinical pilot data. It is important to highlight that no safety concern caused its withdrawal, simply lack of clinical demand in hepatobiliary imaging. The formulation of MnDPDP for clinical use is clearly feasible, as evidenced by its current use in clinical studies (table 1). Similarly, while EVP 1001-1 is not available for routine clinical use, it is currently undergoing clinical trial, again demonstrating the feasibility of manufacture of clinical manganese contrast agents. With promising clinical data emerging, a large scale translation is the essential next step to drive pharmaceutical interest in the production of these agents.

## Conclusions

With a significant body of preclinical data and emerging clinical work in the field, the stage is now set for wider clinical translation of this exciting non-invasive imaging technique. Potential applications are wide-reaching, including direct viability assessment following myocardial infarction, early diagnosis and pathophysiological study of non-ischaemic cardiomyopathy, and in development of novel regenerative heart failure therapies.
